# Contaminant DNA in bacterial sequencing experiments is a major source of false genetic variability

**DOI:** 10.1186/s12915-020-0748-z

**Published:** 2020-03-02

**Authors:** Galo A. Goig, Silvia Blanco, Alberto L. Garcia-Basteiro, Iñaki Comas

**Affiliations:** 1 Institute of Biomedicine of Valencia, IBV-CSIC, St. Jaume Roig 11, 46010 Valencia, Spain; 2 Centro de Investigaçao em Saúde de Manhiça (CISM), Bairro Cambeve, Rua 12, Distrito da Manhiça, 1929 Maputo, Mozambique; 3 ISGlobal, Hospital Clínic - Universitat de Barcelona, Barcelona, Spain; 4 CIBER in Epidemiology and Public Health, Madrid, Spain

## Abstract

**Background:**

Contaminant DNA is a well-known confounding factor in molecular biology and in genomic repositories. Strikingly, analysis workflows for whole-genome sequencing (WGS) data commonly do not account for errors potentially introduced by contamination, which could lead to the wrong assessment of allele frequency both in basic and clinical research.

**Results:**

We used a taxonomic filter to remove contaminant reads from more than 4000 bacterial samples from 20 different studies and performed a comprehensive evaluation of the extent and impact of contaminant DNA in WGS. We found that contamination is pervasive and can introduce large biases in variant analysis. We showed that these biases can result in hundreds of false positive and negative SNPs, even for samples with slight contamination. Studies investigating complex biological traits from sequencing data can be completely biased if contamination is neglected during the bioinformatic analysis, and we demonstrate that removing contaminant reads with a taxonomic classifier permits more accurate variant calling. We used both real and simulated data to evaluate and implement reliable, contamination-aware analysis pipelines.

**Conclusion:**

As sequencing technologies consolidate as precision tools that are increasingly adopted in the research and clinical context, our results urge for the implementation of contamination-aware analysis pipelines. Taxonomic classifiers are a powerful tool to implement such pipelines.

## Background

Whole-genome sequencing (WGS) has enhanced the study of complex biological phenomena in bacteria, such as population dynamics, host adaptation, or outbreaks of microbial infections [1, 2]. In addition, democratization of high-throughput sequencing technologies and continuous improvements in laboratory procedures are also turning WGS into a promising alternative for the clinical diagnosis and surveillance of several pathogenic species [3–5]. Thus, many efforts in the basic and clinical research fields are directed to the improvement of bioinformatic pipelines to ensure the robustness of the conclusions drawn.

Central to many bacterial WGS bioinformatic pipelines is the identification of genetic variants. Incorrect identification of variants can have a major impact on several areas of microbiological research. Applications based on variant analysis include, but are not limited to, phylogenetics [6], phylodynamics and dating [7], genome-wide association studies [8], experimental evolution [9], epidemiological analyses [10], and drug development [11]. Furthermore, the frequency at which each variant is observed in a sample can be used to characterize population genetics processes. Analysis of the allele frequency spectrum enables the study of population dynamics of diversity within a host or co-existence of mixed lineages [12]. In the clinical field, variant analysis at a genomic scale allows the identification of pathogen species and genotypes, distinguish between relapse and superinfections, or prediction of resistance phenotypes and transmission links.

While many factors are taken into account when developing SNP calling pipelines, surprisingly, the potential role of contamination is seldomly considered [13]. However, misinterpretation of contaminated data can lead to draw incorrect conclusions about biological phenomena [14, 15].

Genomic databases are known to encompass contaminated sequences, with assembled genomes that can contain large genomic regions from non-target organisms [16, 17]. Strikingly, a recent study revealed that deposited bacterial and archaeal assemblies are contaminated by human sequences that created thousands of spurious proteins [18]. While the potential impact of contaminants has been considered in fields like metagenomics or transcriptomics, most bacterial WGS analysis pipelines lack specific steps aimed to deal with contaminant data. This situation likely originates from the assumptions that microbiological cultures are mostly free of non-target organisms and that even if present, contaminant sequences are unlikely to map to the reference genomes or are removed using standard filter cutoffs. To date, the extent of contamination and its impact in bacterial re-sequencing pipelines has not been comprehensively assessed.

In this work, we use both real and simulated data to perform a detailed comparison of a standard bacterial mapping and SNP calling pipeline against 2 alternative contamination-aware approaches. First, we implement a taxonomic filter removing contaminant reads that allowed us to assess the extent of contamination and estimate its impact in a dataset comprising 2600 samples of 13 different species from 12 bacterial WGS projects. Second, we compare the performance of this taxonomic filter with a filter based on the similarity of the alignments and evaluate the impact of contamination in 8 WGS projects comprising 1500 samples of *Mycobacterium tuberculosis* (MTB) WGS samples.

We found that contamination events are frequent across bacterial WGS studies and can introduce large biases in variant analysis despite the use of stringent mapping and variant calling cutoffs. Importantly, this is not only true for culture-free sequencing strategies, but also for experiments sequencing from pure cultures. We show that the effect size is not dependent on the amount of contamination and that samples with even low-level contamination can accumulate dozens of errors, particularly for non-fixed SNPs. We demonstrate that removing contaminant reads with a taxonomic classifier allows the implementation of more accurate variant calling pipelines, and provide a validated workflow for WGS analysis of MTB.

## Results

### Contamination is common across WGS studies, even when sequencing from pure cultures

**Fig. 1:**
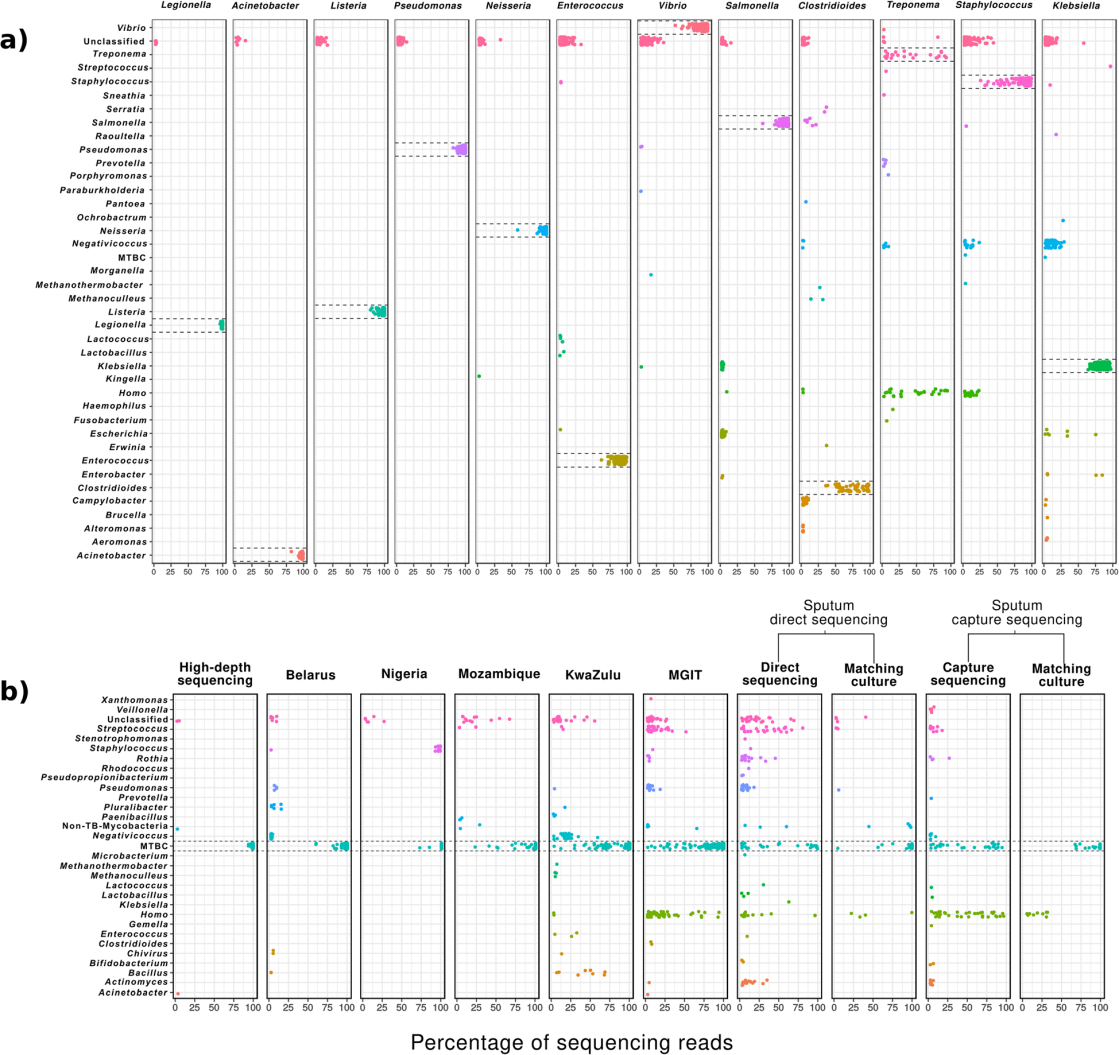
Proportion of sequencing reads for different organisms across 4346 WGS samples from 20 different studies. Each dot represents a sample with a given percentage of sequencing reads coming from the genus indicated in the *y*-axis. Dashed lines highlight the target organism of each study. A 0.3 of vertical jitter was applied for better visualization. Only organisms in a proportion above 2% are shown. **a** Studies of the bacterial dataset. **b** Studies of the MTB dataset. The two *Enterococcus* species analyzed in the bacterial dataset are shown under the same rectangle as they belong to both the same genus and the same study

To assess the extent of contamination across bacterial WGS studies, we taxonomically classified the sequencing reads of 4194 WGS samples from 20 different studies using Kraken, a metagenomic read classifier that has been extensively used and evaluated in the literature. Out of these, 1553 samples corresponded to *M. tuberculosis* sequencing projects, here referred as the *MTB dataset*, and 2641 to 13 other bacterial species, here referred as the *bacterial dataset* (Table 1). According to taxonomic classifications, varying levels of contamination with non-target reads can be found in the different studies (Fig. 1). From the *bacterial dataset*, *Legionella pneumophila*, *Acinetobacter baumannii*, *Listeria monocytogenes*, *Pseudomonas aeruginosa*, and *Neisseria gonorrhoeae* studies showed the expected taxonomic profile from pure culture sequencing, since virtually all the reads are classified in their respective target genus. By contrast, contamination can be clearly found in the rest of studies from this dataset, with an average of 45% of samples per study having less than the 90% of the reads coming from the target organism. The *Treponema pallidum* study represents an extreme case, with its samples having an average of only 40% of reads coming from this organism. This result is expected since in this study the samples were sequenced directly from clinical specimens using a bait capture strategy. However, high levels of contamination can be found in other studies where sequencing is performed from pure cultures (Fig. 1a).

**Table 1:** Studies analyzed

Study name	Publication	Runs analyzed	Sample source	Dataset
Mozambique	Unpublished	138	Clinical isolates	MTB dataset
Kwazulu-Natal	Cohen et al. 2015	433	Single colonies from clinical isolates	MTB dataset
Nigeria	Senghore et al. 2017	73	Clinical isolates	MTB dataset
Belarus	Wollenberg et al. 2017	552	Clinical isolates	MTB dataset
High-depth sequencing	Trauner et al. 2017	63	Clinical isolates	MTB dataset
Sputum-capture sequencing	Brown et al. 2015	58	Clinical respiratory specimens (culture-free sequencing with a bait capture strategy)	MTB dataset
Sputum-direct sequencing	Votintseva et al. 2017	68	Clinical respiratory specimens (direct culture-free sequencing)	MTB dataset
MGIT sequencing*	Pankhurst et al. 2016	168	Early-positive MGIT cultures (liquid)	MTB dataset
*A. baumannii*	Willems et al. 2016	36	Single-colony recultured in broth	Bacterial dataset
*C. difficile*	Stone et al. 2016	54	Pooled single-colony isolates	Bacterial dataset
*Enterococcus*^†^	Tyson et al. 2018	197	Isolates from retail meats	Bacterial dataset
*K. pneumoniae*	Holt et al. 2015	285	Human, animal, and environmental isolates	Bacterial dataset
*L. monocytogenes*	Halbedel et al. 2018	424	Clinical isolates from human	Bacterial dataset
*L. pneumophila*	Timms et al. 2018	48	Pure culture isolates from human and cooling towers	Bacterial dataset
*N. gonorrhoeae*	Yahara et al. 2018	272	Pure culture isolates from human	Bacterial dataset
*P. aeruginosa*	Marvig et al. 2015	445	Clinical isolates from human	Bacterial dataset
*S. aureus*	Aanensen et al. 2016	337	Clinical isolates from 186 hospitals in 21 countries	Bacterial dataset
*S. enterica*	Gymoese et al. 2017	366	Human, animal, and environmental isolates	Bacterial dataset
*T. pallidum*	Pinto et al. 2016	25	Clinical specimens (culture-free sequencing with a bait capture strategy)	Bacterial dataset
*Vibrio*^‡^	Greig et al. 2018	152	Clinical isolates from human	Bacterial dataset

*This study included sequencing samples from non-MTB organisms. We analyzed the 168 reported as MTB by the authors

^†^This study included sequencing samples from 2 species (*E. faecalis and E. faecium*)

^‡^This study included sequencing samples from different *Vibrio* species. We only analyzed the 152 reported as *V. cholerae* by the authors

When looking at the *MTB dataset*, we also observed contamination to be common across studies (Fig. 1b). As expected, direct sequencing from clinical specimens and early positive mycobacterial growth indicator tubes (MGIT), which are inoculated with primary clinical samples, present higher levels of contamination in terms of both the number of samples contaminated and the proportion of non-target reads within them. Common contaminants for these samples comprise human DNA, and bacteria usually found in oral and respiratory cavities like *Pseudomonas*, *Rothia*, *Streptococcus*, or *Actinomyces*, and can constitute virtually all reads in some samples. However, as observed for the *bacterial dataset*, contamination was also detected in studies in which the sequenced DNA came from pure culture isolates. For instance, *Bacillus*, *Negativicoccus*, and *Enterococcus* represented up to 68%, 58%, and 32%, respectively, of different samples from the KwaZulu study. Strikingly, 17 out of 73 MTB samples from the Nigeria study were identified as *Staphylococcus aureus* (92 to 99% of reads). The high-depth dataset was mostly free of contamination, with the exception of two samples for which 3.32% of *A. baumannii* and 2.83% of non-tuberculous mycobacteria (NTM) were identified (representing 795,887 and 920,379 reads, respectively).

### A taxonomic filter to selectively analyze non-contaminant reads

To assess the impact of these contamination events in bacterial WGS analysis, we compared the outcomes in variant calling for each sample before and after removing contaminant reads as classified by Kraken. We refer to this contamination removal methodology as a “taxonomic filter” (detailed in the “Material and methods” section). To assess whether our Kraken setup can be safely used to remove contaminant reads across the analyzed datasets, we first estimated the proportion of reads that can be classified up to the level of species and genus for each organism using simulated sequence reads from the corresponding reference genome. This analysis was performed both including and excluding the reference genomes from the database (Additional file 1: Table S1; Additional file 2: Table S2). For most of the organisms, more than the 99% of the reads could be classified at species level for 250 bp simulated Illumina MiSeq reads (median = 99.35%; 99.07% excluding the reference) with the exceptions of *K. pneumoniae* (97.86%; 97.86% excluding the reference), *S. aureus* (95.01%; 94.98% excluding the reference), and *T. pallidum* (93.54%; 92.96% excluding the reference). Additionally, excluding the reference genome caused *Enterococcus faecalis* to drop from 99.55 to 90.59%. For 100 bp simulated Illumina HiSeq reads, the proportion of reads classified for each organism was lower in every case (median = 98.79%; 97.96% excluding the reference) with a dramatic drop for *T. pallidum* (72.74%; 71.25% excluding the reference), and with the exception of *M. tuberculosis* that remained at 99.98%. At genus level, Kraken was able to classify most of the reads of each organism (median = 99.89% and 99.61% excluding the reference for 250 bp simulations; median = 99.77% and 99.1% excluding the reference for 100 bp simulations) with the exception of *S. aureus* that remained around 95% in every case and *E. faecalis* that was 91.17% after excluding the reference genome. Interestingly, for *T. pallidum*, which showed to be the most difficult organism to classify at species level, 100% of reads were classified at genus level even after excluding the reference genome. Therefore, to safely analyze the effect that contaminant reads have in WGS of the *bacterial dataset*, we applied the Kraken-based taxonomic filter at the genus level (e.g., we removed all non-*Acinetobacter* reads from the *A. baumannii* study).

Second, we scanned all the WGS samples to estimate the maximum proportion of reads Kraken is capable of classify as the target organism in real samples (Additional file 1: Table S1). In most cases, there was at least one sample per bacteria that could be classified as well as the reference genome (median difference between real and simulated sequencing of 1% at species level and 0.35% at genus level). The higher difference was observed for *T. pallidum* for which the maximum number of reads classified in a real WGS sample at genus level was 94.75%. This most likely reflects that sequencing directly from clinical specimens usually produces contaminated samples.

Third, a fraction of reads that actually come from the target organism may be misclassified, and thus, such reads would be incorrectly removed by the taxonomic filter. In order to estimate the magnitude of this error in our analysis, we used Bracken to calculate the fraction of misclassified reads that were expected to actually belong to the target organism. Overall, the proportion of reads incorrectly eliminated by the taxonomic filter as estimated by Bracken was very low (median = 0.11%, IQR = 1.32%). This proportion varied between different organisms (Additional file 3: Table S3). For example, whereas for several organisms like *A. baumannii*, only 0.07% of the reads eliminated by the taxonomic filter are estimated to actually belong to *Acinetobacter*, in the case of *K. pneumoniae*, in which a high proportion of reads remain unclassified or are assigned to *Negativicoccus massiliensis*, 3.65% are estimated to actually belong to *Klebsiella.*

Additionally, it can be argued that highly contaminated samples are likely to be detected by basic sequencing quality controls regardless of the implementation of specific contamination-control analysis. In contrast, contaminating reads would likely pass unnoticed when enough data from the target organism is produced and quality parameters like sequencing depth or genome coverage meet certain criteria. Furthermore, the measures of the impact of contamination in SNP analysis may be biased by including highly contaminated data. Thus, for the following analyses, we discarded samples with contamination higher than 50% or depths lower than 40× (only a minimum of 20× was required for *T. pallidum*, see the “Material and methods” section for a further explanation). From the initial 2641 samples of the *bacterial dataset*, 2233 met these criteria (408 had less than 40× depth and 16 had more than 50% of contamination (Additional file 4: Table S4)).

### Contamination impacts bacterial WGS analysis

**Fig. 2:**
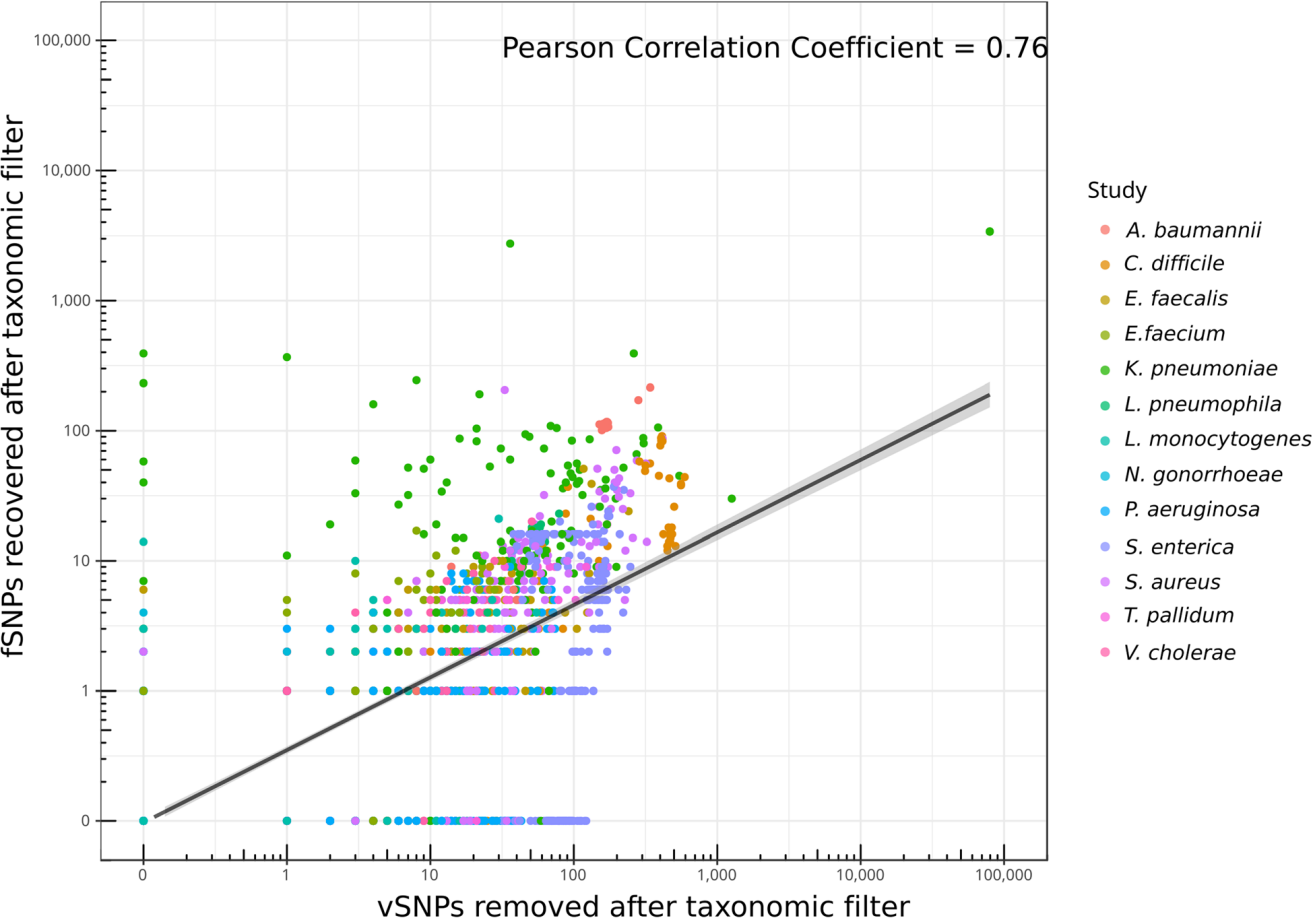
Correlation between the number of vSNPs removed and the number of fSNPs recovered after contamination removal with the taxonomic filter

The expected effect of mapped contaminant reads is to produce mixed calls, leading to the identification of false positive variable SNPs (vSNPs). These false positive calls would alter the frequencies calculated at a given position, which might also produce false negative fixed SNPs (fSNPs) by lowering the frequency below the required cutoff to call fixed variants (90% frequency in this work). The Pearson correlation coefficient between removing vSNPs and recovering fSNPs was of 0.76 (Fig. 2). However, not all the contaminant reads are expected to affect positions with fSNPs, and in fact, for 405 samples (18%), the taxonomic filter removed the false positive vSNPs without affecting any fSNP. Similarly, in 38 samples (3%), we observed the recovery of at least 1 false negative fSNP without removal of vSNPs. Notably, we did not observe a correlation between the number of vSNPs removed and the degree of contamination of a sample (Pearson’s correlation coefficient = − 0.06) (Table 2). This result suggests that the impact in variant analysis is highly dependent on the identity of both the contaminant and the target organisms, rather than the number of contaminating reads.

**Table 2:** Effect of applying the taxonomic filter in the variant analysis of samples of the *bacterial dataset*

Study	Mean percentage of target organism (%)	Mean number of vSNPs removed (median; IQR)	Mean number of fSNPs recovered (median; IQR)	Pearson’s correlation coefficient between removal of vSNPs and recovery of fSNPs	Pearson’s correlation coefficient between removal of vSNPs and percentage of target organism
*A. baumannii*	97.30	89 (43; 165)	57 (10; 113)	0.99	0.25
*C. difficile*	76.74	299 (397; 379)	27 (16; 32)	0.45	0.23
*E. faecalis*	89.96	30 (19; 33)	4 (3; 5)	0.65	− 0.13
*E. faecium*	94.38	9 (5; 10)	3 (2; 5)	0.47	− 0.45
*K. pneumoniae*	84.38	549 (62; 112)	73 (13; 41)	0.76	− 0.44
*L. pneumophila*	99.06	12 (0; 8)	3 (0; 1)	0.99	− 0.63
*L. monocytogenes*	98.42	2 (0; 1)	0 (0; 0)	0.49	− 0.43
*N. gonorrhoeae*	99.17	0 (0; 0)	0 (0; 0)	0.34	− 0.09
*P. aeruginosa*	97.43	9 (2; 14)	1 (0; 1)	0.50	− 0.11
*S. enterica*	95.01	97 (91; 87)	7 (6; 12)	0.14	0.02
*S. aureus*	91.42	50 (22; 50)	9 (3; 9)	0.54	− 0.10
*T. pallidum*	39.75	45 (34; 52)	6 (5; 4)	0.63	− 0.48
*V. cholerae*	91.32	9 (5; 16)	2 (1; 3)	0.76	− 0.56

**Fig. 3:**
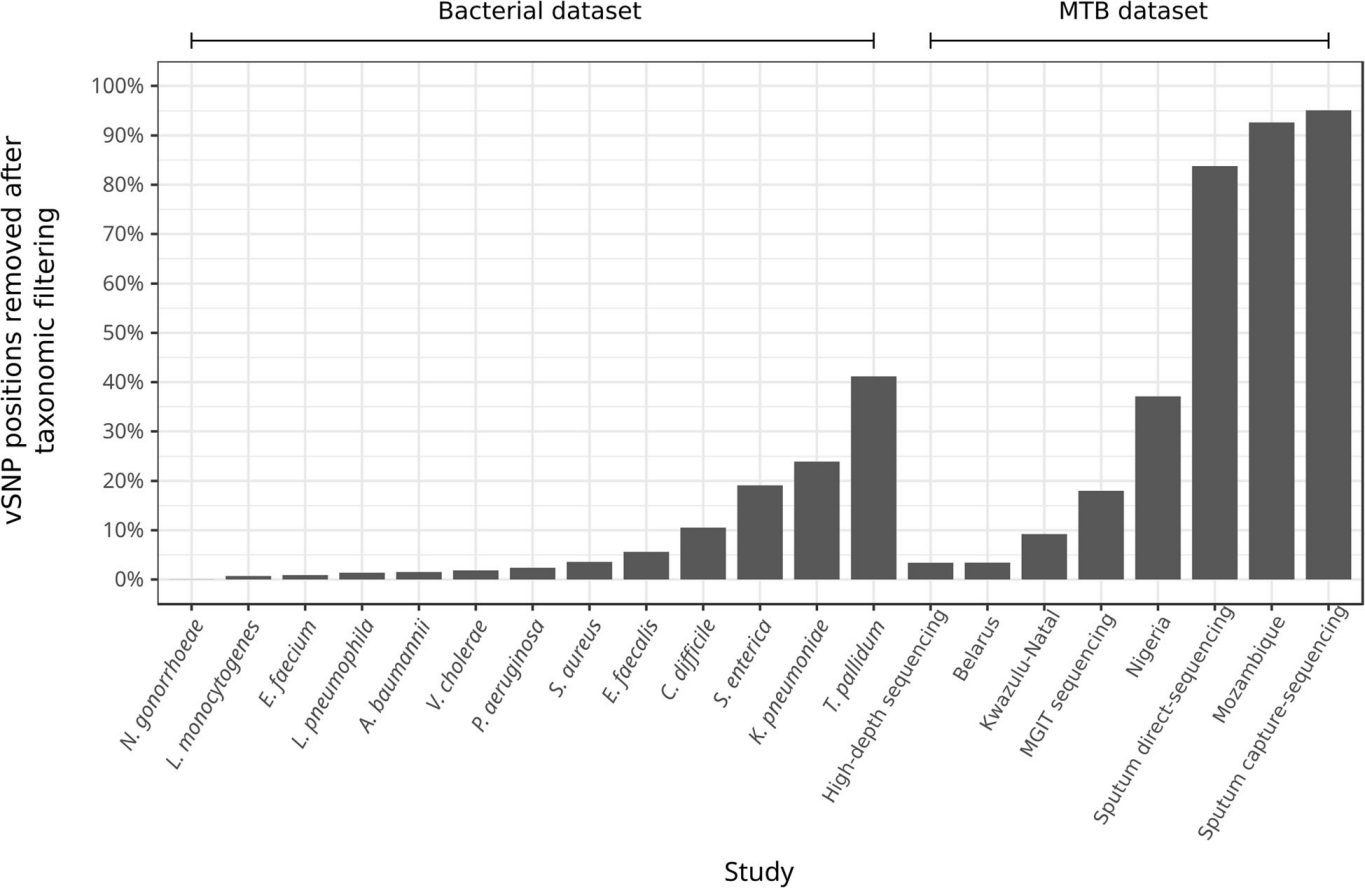
Fraction of polymorphic positions with vSNPs removed after applying the taxonomic filter

Overall, the impact of removing contaminant reads on vSNP and fSNP inference depended heavily on the species considered. For example, virtually, no change was observed for *N. gonorrhoeae* samples (Table 2, Fig. 3) while a mean number of 89 vSNPs were removed and 57 fSNPs recovered for *A. baumannii* samples. The greatest change was observed for *K. pneumoniae*, *S. aureus*, and *Salmonella enterica* datasets. However, in these cases, the impact of contaminant reads might be overestimated due to the incorrect elimination of target reads by the taxonomic filter. In many WGS applications, genetic variants are not analyzed on a sample basis but across the entire dataset. We therefore evaluated the impact of contaminant reads on polymorphic positions called across datasets. On average, the total number of polymorphic positions was reduced by 1.51% for fSNPs (range 0–6%) and 8.67% for vSNPs (range 0–41%) (Fig. 3, Additional file 5: Table S5).

We also observed a small proportion of fSNPs to be systematically removed by the taxonomic filter (median = 0.2% of fSNPs, ranging from 0 to 5.6% between studies; Additional file 6: Table S6). Those positions can be considered false negatives introduced by the pipeline, including inconsistencies of the mapping software, and the inability of Kraken to classify a small proportion of reads disregarding their similarity to the reference genome. We observed this to occur mostly in regions of low coverage that could be the result of, for instance, hard-to-map regions like repetitive elements or different strains coexisting in the same sample one of which has a deletion. In such regions, eliminating only one read can lead to greater differences in frequencies making the position fall below the required thresholds to call a fSNP (Additional file 7: Figure S1). Most of the fSNPs incorrectly removed after the taxonomic filter were caused by the inability of Kraken to classify some reads up to the level of genus. This behavior is a known limitation of the taxonomic classifiers for conserved regions among bacteria*.* However, in many cases, the incorrectly eliminated sequences corresponded to reads mapped with 100% identity to the reference genome and surrounded by other sequences that were classified, even at the level of species, despite having several SNPs. This is probably due to the fact that for some reads, the *k*-mers in which they are decomposed do not allow Kraken to classify beyond a given taxonomic level, disregarding the sequence diversity. However, since the immediate contiguous reads can be correctly classified, this bias is normally compensated by the sequencing depth.

Unexpectedly, we also observed an inconsistency of the mapping software (bwa-mem) to be responsible for a small fraction of the SNPs that are either removed or recovered after applying the taxonomic filter. In these cases, we observed that the number of supporting reads at a given position differed in one read despite the fact that none of the reads mapping to that position were classified as contaminant. Surprisingly, we observed this to be the result of the exact same read, mapping to the same genomic position, but with different qualities for the filtered and non-filtered fastq files. The fact that the fastq files are different (because some reads are removed in the filtered fastq) makes bwa to produce different results for a small number of reads (between 1 and 6 in our tests) that are filtered because of the mapping quality cutoff (60). We confirmed this behavior by randomly sorting and mapping different times a set of fastq files (tested versions 7.10, 7.12, and 7.17). This is likely due to the heuristics implemented in the seed-and-extension algorithm of bwa-mem.

Interestingly, we observed *Negativicoccus massiliensis* to be present at high proportions in several datasets. Analyzing a subset of these reads using the NCBI blast utility revealed that they present nucleotide similarity with eukaryotic organisms (e.g., *Cyprinus carpio* and *Plasmodium vivax*). Despite being contaminant reads, their classification as *N. massiliensis* is clearly artifactual, probably due to the absence of eukaryotic organisms in our database other than human. Similarly, Kraken left a high proportion of reads unclassified in many samples. This could be mainly due to either the absence of the organism from the database, or the sequences that Kraken cannot classify up to the level of genus, for instance when analyzing organisms with high genetic diversity. Indeed, when using the NCBI blastn to search a random subset of unclassified reads in the non-redundant database (nr), we observed three main patterns: reads that either did not produce significant matches with any organism, or came from eukaryotes not present in our Kraken database; reads that produced partial alignments with many different taxa; and reads that produced good alignments, even with the target organism, but having alignment identities below 90%, what makes Kraken unable to find exact matches of 31 bp.

### Implementation of a contamination-aware analysis pipeline: *Mycobacterium tuberculosis* as a test case

**Fig. 4:**
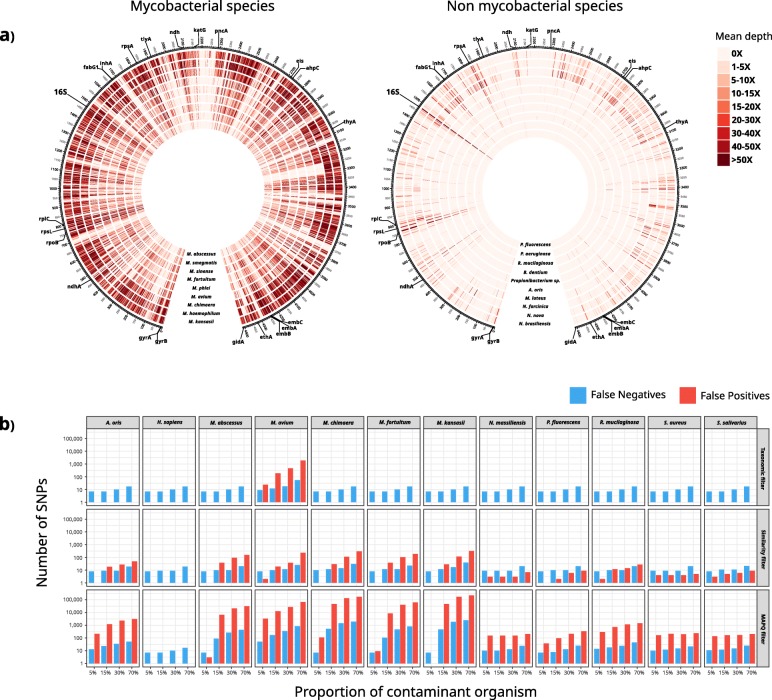
Mapping of non-MTB reads across the MTB reference genome impacts variant calling. **a** Mean sequencing depth along the MTB reference genome across 1000 bp windows when mapping 1500,000 simulated reads of non-tuberculous mycobacteria species and organisms other than mycobacteria (OTM). For OTM, the 10 organisms that produced higher sequencing depths are shown. **b** Number of false positive vSNPs and false negative fSNPs (note log scale) in samples in silico contaminated with different proportions of non-MTB organisms when following 3 different analysis pipelines (taxonomic filtering, similarity filtering, and a standard pipeline including a mapping quality filter (MAPQ 60))

The analysis of the *bacterial dataset* revealed that contaminant reads can have a major impact in bacterial diversity estimations. It is clear, however, that a unique approach might not be appropriate for all organisms and that implementations of contamination-aware pipelines must take into account the genetic particularities of each organism and rely on comprehensive validations. Following the analysis of the *bacterial dataset*, we implemented and extensively evaluated two contamination-control approaches on top of a specific analysis pipeline for *M. tuberculosis*, which is the pathogen our laboratory is focused on. We tested the Kraken-based taxonomic filter at species level (*Mycobacterium tuberculosis* complex) and a similarity filter that removes read mappings with identity and length lower than 97% and 40 bp, respectively. We tested both approaches using simulated and real sequencing runs. In first instance, we used simulated experiments to evaluate how non-MTB reads are mapped to the MTB reference genome and quantify the false positive and negative SNPs that arise consequently. We mapped simulated sequencing samples of 45 organisms to the MTB reference genome, including oral and respiratory microbiota, clinically common non-tuberculous mycobacteria, and human reads. As expected, conserved genes like the 16S, *rpoB*, or the tRNAs constitute hotspots where contaminant sequences are frequently mapped. However, non-MTB alignments are not only produced in these regions but across the reference genome (Fig. 4a). This is dependent on the phylogenetic relationship of the contaminant organism to the one being studied. Non-tuberculous mycobacteria represent the best example of this, as their read mappings can produce high sequencing depths along the MTB reference genome. Human reads, which are a frequent concern in clinical studies, did not produce any alignments at all.

Next, we evaluated the performance of the taxonomic filter and the similarity filter using in silico contaminated samples. Although both approaches reduced the number of non-MTB mappings, the taxonomic filter showed the best performance, eliminating all non-MTB alignments with the only exception of a proportion of *Mycobacterium avium* reads. Accordingly, the number of false positive vSNPs due to contaminants was reduced with both methods, but in the case of the taxonomic filter, almost all erroneous SNP calls were eliminated (Fig. 4b). Only contamination with *M. avium*, a closely related bacteria, compromised its performance. Nonetheless, the errors observed were notably lower than when only using a mapping quality threshold (60 in this work). For example, when a 5% of *M. avium* was present, the 3325 false positive vSNPs and 51 false negative fSNPs identified were reduced to 24 and 9, respectively, after applying the taxonomic filter. The few false negative fSNPs observed in Fig. 4b which are systematic between all methods were due to some positions next to hard-to-map regions that do not pass the coverage cutoffs required to call a fSNP in contaminated samples.

Remarkably, even a 5% of contaminating reads can introduce a large number of false positive vSNPs. As expected, the erroneous calls produced by such small contamination fall mainly in conserved regions. However, in agreement with the results shown in Fig. 4a, spurious SNPs can be called across the genome (Additional file 8: Figure S2). Importantly, it is precisely because many of the contaminant alignments are produced in conserved genes that we predicted false antibiotic resistances, including well-known mutations to first line drugs in MTB treatment (Additional file 9: Table S7).

We also evaluated whether these filters systematically remove sequencing reads from particular genomic regions leading to biases produced by the methodology itself. To do so, we analyzed the mean sequencing depth obtained across the genome, before and after applying the filters, for all the samples of the *MTB dataset* that have less than 1% of contamination (984 samples; 78% of the samples analyzed). Importantly, we observed the taxonomic filter to systematically remove sequencing reads coming from the 16S gene due to the inability of Kraken to classify many reads coming from this gene up to the level of species. However, for the rest of the genome, it showed an excellent performance, with virtually no differences in depth, even for conserved regions like the *rpoB* gene (Additional file 10: Table S8). On the contrary, the similarity filter produced a systematic decrease in depth across the genome. In the 97% of the genome, the sequencing depth was reduced more than 1×, with several regions showing larger decreases (Additional file 11: Table S9).

### Impact of contamination in clinical WGS samples of *Mycobacterium tuberculosis*

After evaluating the performance of the taxonomic and similarity filters, we used them to remove contaminants in a dataset comprising 1553 MTB WGS samples from 8 different studies. As done for the *bacterial dataset*, we only analyzed samples with at least 50% of reads classified as *Mycobacterium tuberculosis* complex and 40× of median sequencing depth (20× for direct sequencing from clinical specimens) to discard heavily contaminated samples (1267 samples, 81.6% of the *MTB dataset*).

Given that the taxonomic filter showed to be extremely conservative with all genomic positions except the 16S gene, we discarded from the following analyses any SNP called in this region (*rrs*, *rrl*, *rrf*). Therefore, the differences observed in variant analysis when applying this filter can be attributed to noise introduced by contamination. In accordance, we expected no differences in variant calling in samples not affected by contaminants. When analyzing real WGS MTB samples with the taxonomic filter, we observed no variant change for 788 samples (62% of the samples analyzed). Importantly, this agreement was true for samples with low-level contamination (less than 1%) but also for samples with higher number of contaminant reads (up to 31%), probably from organisms genetically distant to MTB. Overall, the numbers of SNPs either removed or recovered after applying the taxonomic filter were independent of the level of contamination of a sample (Pearson’s correlation coefficient = 0.03). Altogether, these results strongly suggest that the changes observed in variant analysis after applying the taxonomic filter can be attributed to noise introduced by contaminants rather than a methodological bias. On the contrary, the similarity filter always removes variant positions even for the 984 samples with 99% of MTB. This is in agreement with the higher rate of false negatives observed in the in silico experiments.

**Fig. 5:**
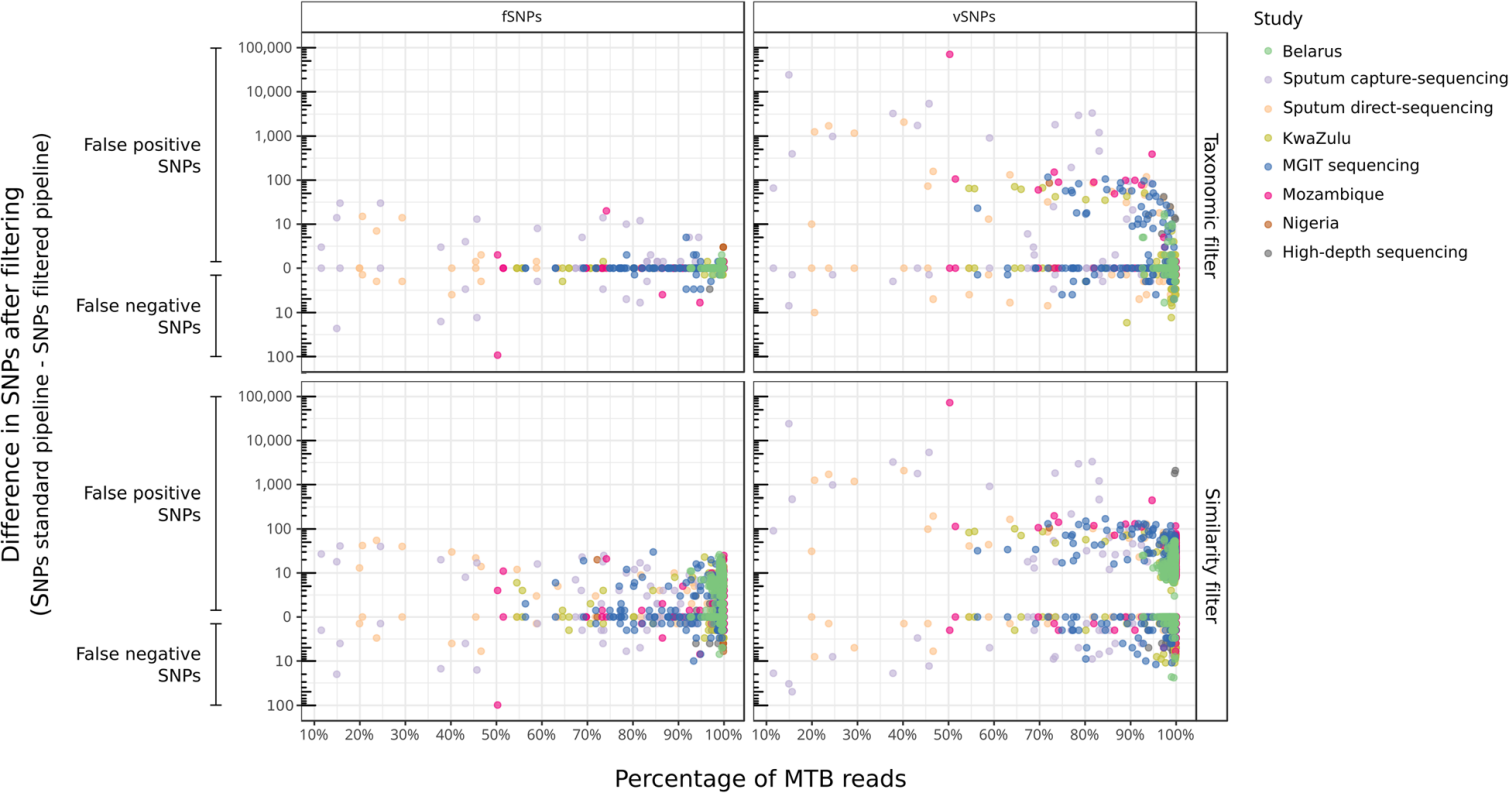
Differences in SNP calling in samples of the MTB dataset between a standard pipeline and the two contamination-control methodologies tested

Mapped contaminant reads introduce new variants that alter the allele frequencies. After applying the taxonomic filter, we observed a mean change of 42% allele frequency (median = 41%; IQR = 36%). As shown in Fig. 5, the main consequence of these alterations is the introduction of many false positive vSNPs, even for samples with contamination levels as low as 5%. However, altering allele frequencies can also lead to call false negative vSNPs, and false positive and negative fSNPs. Among the 38% of samples for which at least 1 change was observed, the taxonomic filter removed on average 761.7 vSNPs (median = 18) and 4 fSNPs (median = 1), and recovered 1.7 vSNPs (median = 1) and 5.9 fSNPs (median = 2). On average, the total number of polymorphic positions within each study was reduced by 0.4% for fSNPs (range 0–2%) and 43% for vSNPs (range 3–95%) (Fig. 3, Additional file 5: Table S5). Applying the similarity filter removed on average 129.1 vSNPs (median = 20) and 6.1 fSNPs (median = 5) and recovered 2.6 vSNPs (median = 2) and 2.3 fSNPs (median = 2).

Sequencing directly from clinical specimens is subject to greater alterations in variant analysis (Fig. 5) since this strategy usually yields highly contaminated samples and limited sequencing depth. In these cases, the SNP frequencies are more sensitive to contaminant reads since only few reads can be responsible for a shift in the frequencies that make a position to fall below or above the required thresholds to call a variant (Additional file 7: Figure S1). However, a high sequencing depth does not guarantee an analysis safe of errors either. This effect can be observed in the high-depth sequencing study, a work based on low-frequency variant analysis from samples with more than 1000× sequencing depth. In this study, 7 samples out of 63 showed changes in the SNP analysis after applying the taxonomic filter. On average, 16.9 false positive vSNPs were removed (ranging from 2 to 42 vSNPs), and for 1 sample, 3 false negative fSNPs and 2 vSNPs were recovered. Remarkably, no strong contamination was detected for these samples (with MTB ranging from 96.86 to 99.84%). For instance, in a sample with as much as 99.84% of MTB, the taxonomic filter removed 13 false positive vSNPs in 12 different genes across the genome.

## Discussion

In this work, we analyze more than 4000 WGS samples from 14 different pathogenic bacterial species to evaluate the extent and impact of contamination in bacterial WGS studies. We show that presence of sequencing reads from contaminating organisms is frequent, even when sequencing is performed from pure culture isolates (Fig. 1). Beyond inappropriate laboratory practices, there are several potential sources of contamination which depend on different factors such as the type of sample processed and its origin, or the protocols followed for culture, DNA extraction, and sequencing. For instance, Salter et al. demonstrated that contaminating DNA in laboratory reagents can critically impact microbiome analysis from low-biomass samples [19]. Culture-free sequencing approaches for unculturable or slow-growing pathogens, such as *T. pallidum* or MTB, entail the presence of high amounts of contaminating DNA from the host organism. Other sources unrelated to sample handling are also possible. For example, the *S. aureus* samples supposed to be MTB from the Nigeria study are most likely an error during data submission to the genomic repository. Regardless of the source of contamination, the shared consequence is the presence of non-target reads in the sequencing files that might impact the results of genomic analysis.

We evaluated such an impact and demonstrate that contaminant reads suppose a pitfall in re-sequencing pipelines, since they are unexpectedly frequent and can have major implications in variant analysis, which is the foundation of many genomic analyses. As expected, contamination is a major issue when sequencing DNA that has not been extracted from pure cultures or single colonies, as is often the case for clinical specimens. However, we show that experiments sequencing from pure cultures are not necessarily free of contamination, and that using standard mapping quality parameters is not enough to deal with contaminant reads. Therefore, bioinformatic pipelines assuming that all the reads successfully mapped are from the target organism might lead to a biased variant analysis. We show that the errors introduced by contamination are very variable among different studies, (Table 2; Fig. 3; Fig. 5), which differ not only on the organism being sequenced but also on the sampling source and laboratory protocols. For example, in the *T. pallidum* study, where samples are heavily contaminated, very few differences are observed in the variant analysis. This stems from the fact that most of the contamination in this study comes from human reads, unlikely to map to the *T. pallidum* genome. On the contrary, for the *L. pneumophila* dataset, a sample with 96.27% of *Legionella* had 79 vSNPs and 5 fSNPs removed, and 17 fSNPs recovered after filtering a 3% of unclassified reads. According to the NCBI blast, a fraction of those reads was from *Legionella spiritensis.* The downstream relevance, however, is not directly proportional to the absolute number of erroneous SNPs and frequencies, but to what that errors mean for each organism. For example, for organisms with low genetic diversities, like in the case of MTB, a change in few fSNPs can have major implications in epidemiology studies since transmission cutoffs vary between 5 and 12 fSNPs [20]. This is also true when predicting drug resistance, particularly considering that many drug resistance-associated genes are conserved among bacteria and hence more prone to recruit contaminant mappings. Likewise, the higher impact observed for vSNPs, both in terms of absolute numbers and frequencies, can have large implications in those applications based on the analysis of the allele frequency spectrum, for example, when studying complex traits in bacterial populations. For instance, vSNPs are analyzed to determine heteroresistance to antibiotics [21], within host diversity of pathogens [22], host adaptation of bacteria [23], and even to delineate between patient transmission of pathogens [24]. While not specifically tested in our analysis, our results also have obvious implications in other applications which highly depend on the variation detected (e.g., cgMSLT typing) or when contaminant reads are incorporated in de novo assemblies [18].

The main limitation of our study is that we have based our estimations on the taxonomic classifications of Kraken. However, taxonomic classifiers are known to misclassify a proportion of reads that are thus incorrectly identified as contaminants. We took into account several considerations to control for the potential biases in our analysis. Whereas Kraken is computationally expensive, its performance has been demonstrated in several studies to rank among the best up to date [25–27]. Secondly, since distinguishing between closely related species may be difficult, to be conservative, we performed the taxonomic filter at the genus level instead of species. Additionally, we estimated the error introduced by Kraken in our own setting, particularly regarding unclassified and misclassified reads, and showed that the error rate was very low (Additional file 1: Table S1; Additional file 2: Table S2; Additional file 3: Table S3), in agreement with published data. Despite these measures, we might have under- or overestimated the impact of contaminant reads in some cases. For example, by removing non-target reads at the level of genus, we might have underestimated the impact of potential contamination, given that contaminant reads from the same genus (but different species) are more likely to map to the reference genome and thus impact variant analysis. Our analysis also showed that Kraken might have overestimated the number of contaminant reads in some datasets due to, for example, exchange of genetic material between species (*K. pneumoniae*, *S. aureus*, *S. enterica*) or the absence of enough genetic diversity in the database.

Altogether our results show that contaminant reads in re-sequencing experiments are frequent and can greatly bias variant analysis at a genomic level. However, based on our results, it is clear that different settings will require different contamination-control strategies that take into account the genetic particularities of each organism. Whereas the taxonomic filter we propose seems to perform well in many situations, in the case of highly diverse bacteria, other approaches might be better suited. For instance, coverage information and *k*-mer frequencies [28, 29] can be used to distinguish between target and contaminant reads when these are present in significantly different proportions. Similarly, detecting cross-contamination with strains of the same species is challenging and requires specific strategies. These strategies can include detection of vSNPs at lineage-defining positions, calculation of biased allele ratios [30], or Bayesian statistical modeling [31].

Importantly, different implementations of such strategies should be extensively evaluated and validated. Here, we provide such an evaluation for the pathogen our laboratory is focused on: *M. tuberculosis*. In addition to the taxonomic filter, we evaluated a second contaminant filtering approach based on the similarity of the read alignments. In this case, the Kraken-based taxonomic filter clearly outperformed the similarity filter what is probably true for other organisms with representative genomes in the databases and moderate genetic diversities (Fig. 4, Fig. 5, Additional file 1: Tables S8 and S9, and Figure S1).

The analyses for MTB reveal a large number of variants introduced by contaminants with downstream consequences when calling vSNPs and fSNPs as well as the wild type. Remarkably, we show that contamination can introduce substantial errors in samples that could be considered “pure” or with high sequencing depths, implying that contamination-aware pipelines will be needed in any circumstance.

Contamination has been recognized as a major source of error in genome assemblies and other fields like metagenomics [16, 19]. However, the role of contamination in re-sequencing pipelines is usually neglected. Whereas some groups are already aware of this issue, most bacterial re-sequencing pipelines are still lacking contamination-control strategies or, if any, these are rarely detailed in published works. Based on our findings, we call for the inclusion of contamination control as a basic quality parameter and the use of validated contamination-aware pipelines in any bacterial WGS study. These analyses pipelines should be capable of, at least, reporting the contaminated samples and their contaminants to be later interpreted by the researcher. Ideally, they should be able to produce accurate results regardless of the extent of contamination of a sample. Pipelines capable of accurately analyzing contaminated WGS data will soon become essential, since the improvement of laboratory protocols allows the sequencing of an increasing number of bacterial species directly from clinical specimens [32, 33]. In this work, we provide a highly accurate contamination-aware pipeline for MTB WGS analysis that will be extremely helpful in the upcoming studies and clinical applications sequencing MTB directly from respiratory samples.

## Material and methods

### Datasets analyzed from bacterial WGS studies

In order to detect contamination through different studies and evaluate its impact in bacterial WGS experiments, we analyzed WGS runs from 20 different studies. We considered studies that have been published recently and for which Illumina sequencing reads were already available. The datasets comprised 8 MTB studies and 12 studies of other 13 relevant pathogenic species. Nineteen of these datasets were publicly available beforehand [34–52]. To include a dataset generated in our laboratory, we sequenced 138 MTB samples from Mozambique in the Illumina MiSeq platform. A total of 4194 Illumina runs were analyzed, comprising 1553 MTB samples (*MTB dataset*) and 2641 samples from the rest of organisms (*bacterial dataset*) (Table 1).

### Whole-genome sequencing of MTB samples from Mozambique

DNA extractions were performed in heat-inactivated samples of MTB Löwenstein-Jensen cultures with an automated DNA extraction platform (NucliSENS EasyMag; bioMérieux). Sequencing libraries were prepared with Nextera XT DNA Library Preparation Kit v3 (Illumina, San Diego, CA) following the manufacturer’s instructions. Whole-genome sequencing was performed on the Illumina MiSeq instrument with 2X300bp paired-end reads.

### Contamination assessment using Kraken

In order to assess contamination in each dataset, sequencing reads were taxonomically classified using Kraken [53] with a custom database comprising all sequences of bacteria, archaea, viruses, protozoa, plasmids, and fungi in RefSeq (release 78), plus the human genome (GRCh38, Ensembl release 81). Kraken classifications and Kraken database setup were performed with default parameters. Bracken [54] was used to estimate the number of misclassified reads that could be reassigned to the target organism.

### Analysis pipeline

To analyze WGS data, we used a general analysis pipeline for read mapping and variant calling. In summary, bases with an average quality below 25 in a 20-bp window were trimmed and reads shorter than 50 bp were filtered. Sequences were then mapped to the reference genome of each organism using bwa-mem [55]. We used as reference genomes those used by the authors in their respective manuscripts when specified and otherwise the representative genome of RefSeq (Additional file 12: Table S10). For MTB samples, we used the genome of the inferred most recent common ancestor of the *Mycobacterium tuberculosis* complex. Alignments with mapping qualities (MAPQ) below 60 were removed. Variants were then called and filtered using two different set of parameters to call fixed SNPs (fSNPs) and variable SNPs (vSNPs). The cutoffs to call fSNPs were minimum depth of 20 reads, with the variant observed in at least 20 reads; average base quality of 25; *p* value cutoff of 0.01, observed in both strands; and minimum frequency of 90%. The cutoffs to call vSNPs were minimum depth of 10 reads, with the variant observed in at least 6 reads; average base quality of 25; *p* value cutoff of 0.01, observed in both strands; and minimum frequency of 10%. We also removed SNPs near indels in a window of 4 bp. For MTB samples, we used an additional annotation filter to remove SNPs in repetitive and mobile regions. Additionally, to call fSNPs, we used a density filter removing SNPs within high-density regions (allowing a maximum of 3 SNPs in 10 bp windows). This filter is commonly used in MTB WGS data since it is not expected to observe many contiguous variants given the extremely low genetic diversity of this species.

We compared this general analysis pipeline with two approaches for contamination removal. The “taxonomic filter” consisted of the removal of contaminant reads after the trimming step, prior to mapping. For MTB samples, we removed those reads classified by Kraken as any species other than *Mycobacterium tuberculosis* complex. In the case of organisms other than MTB, to be conservative, we removed the reads classified as any organism other than the target at the level of genus, keeping also those sequences classified as phages of those organisms. For MTB samples, we additionally evaluated a method consisting in a custom *similarity filter*. We tested several combinations that filtered the alignments based on their similarity, length, and mapping quality (Additional file 13: Figure S3). The similarity filter finally consisted in the removal of alignments with length, identity, and quality below 40 bp, 97%, and 60, respectively.

We only considered for analysis samples with more than the 50% of the target organism and with a median sequencing depth of at least 40×. In the case of studies performing WGS directly in clinical samples (sputum-capture sequencing, sputum-direct sequencing, and *Treponema* studies), we analyzed those samples that had at least 20× of median coverage, since this type of sequencing is expected to sequence samples with lower coverages and high proportions of non-target reads.

### Generation of simulated datasets

We used the reference genome of each organism to generate simulated sequencing samples using ART [56]. We generated paired-end sequencing data of 250 and 100 bp using the error profiles of Illumina MiSeq (--ss MSv3) and Illumina HiSeq (-ss HS20) platforms, respectively. This allowed us to estimate the proportion of reads that cannot be classified by Kraken up to level of genus and species for each organism. The same approach was used to generate sequencing runs of different bacterial contaminants commonly observed in MTB WGS samples (see below). The command line used to generate the sample was as follows: art_illumina -ss [MSv3 | HS20] --rcount 2000000 -l 250 --mflen 800 --paired --minQ 25 -s 300

### Evaluation of the impact of contamination and methodology validation

We generated simulated sequencing of the MTB reference genome, the human genome (GRCh38, Ensembl release 81), and 44 different non-MTB bacterial species (Additional file 14: Table S11). This allowed us to perform two kinds of experiments (mapping of non-MTB reads to the MTB reference genome and analysis of mock contaminated samples) as explained further below.

In order to inspect which regions of the reference genome are susceptible of recruiting non-MTB reads, we mapped the simulated reads and then measured the mean sequencing depth across the genome in 1000 bp windows. To assess whether false positive SNPs and drug resistance predictions are produced by these non-MTB mappings, we generated mock contaminated samples by mixing sequencing reads of the reference genome with different proportions (5%, 15%, 30%, and 70%) of other organisms corresponding to 12 common contaminants identified in the *MTB dataset.* Therefore, any SNP identified when analyzing these samples would be a false positive, attributable to contamination.

In addition, we mapped these mock samples to a modified version of the reference genome where we introduced random mutations each 100 bp, and all the drug resistance conferring mutations described as “high confidence” in the PhyResSE catalog [57]. Therefore, any of the introduced SNPs that were undetected when analyzing these samples would be false negatives attributable to contamination.

## Supplementary information

**Additional file 1: Table S1.** Evaluation of the performance of Kraken classifying reads at genus and species level for the reference genomes and among all samples of the studies analyzed.

**Additional file 2: Table S2.** Evaluation of the performance of Kraken classifying reads at genus and species level for the reference genomes and among all samples of the studies analyzed after excluding the reference genomes from the Kraken database.

**Additional file 3: Table S3.** Bracken estimation of the proportion of misclassified and unclassified reads that actually belong to the target organism.

**Additional file 4: Table S4.** Samples of the bacterial dataset with less than 50% of target organism.

**Additional file 5: Table S5.** Difference in the number of variant positions within a dataset between the basic and the taxonomic-filtered pipeline.

**Additional file 6: Table S6.** Proportion of fSNPs removed per sample in the *bacterial dataset. (DOCX 6 kb)*

**Additional file 7: Figure S1.** Effects of contaminations and taxonomic filtering in variant calling.

**Additional file 8 :Figure S2.** Contaminations can lead to incorrect calls across the *M. tuberculosis* genome.

**Additional file 9: Table S7.** Evaluation of false drug resistance predictions in mock contaminated samples.

**Additional file 10: Table S8.** Genomic regions (1000 bp windows) with a coverage decrease greater than 1X after taxonomic filtering in 984 samples of the *MTB dataset* with more than 99% of reads classified as MTB.

**Additional file 11: Table S9.** Top ten genomic regions (1000 bp windows) with greater coverage decrease after applying the similarity filter in 984 samples of the *MTB dataset* with more than 99% of reads classified as MTB.

**Additional file 12: Table S10.** Reference genomes of the *bacterial dataset*.

**Additional file 13: Figure S3.** Implementation of the similarity mapping filter.

**Additional file 14: Table S11.** Non-MTB species included in the simulated sequencings to evaluate the impact of contaminations in MTB WGS samples.

## Data Availability

All data generated or analyzed during this study are included in this published article, its supplementary information files, and publicly available repositories. Whole-genome sequencing data from Mozambique isolates generated in our laboratory is available at the European Nucleotide Archive under the accession PRJEB27421 [58]. The inferred most recent common ancestor genome of the *Mycobacterium tuberculosis* complex is available at Zenodo [59].
