# Supplementary material for: Contaminant DNA in bacterial sequencing experiments is a major source of false genetic variability
Source: BMC Biol. 2020 Mar 2;18:24. doi: 10.1186/s12915-020-0748-z (PMC7053099; doi:10.1186/s12915-020-0748-z)
Supplement: Supplementary file 1 — Additional file 1: Table S1. Evaluation of the performance of Kraken classifying reads at genus and species level for the reference genomes and among all samples of the studies analyzed. [file 12915_2020_748_MOESM1_ESM.docx]

**Table S1.** Evaluation of the performance of Kraken classifying reads at genus and species level for the reference genomes and among all samples of the studies analyzed.

| **Organism** | **Reads classified as target species (Illumina MiSeq; 250bp)** | **Reads classified as target genus (Illumina MiSeq; 250bp)** | **Reads classified as target species (Illumina HiSeq; 100bp)** | **Reads classified as target genus (Illumina HiSeq; 100bp)** | **Maximum proportion classified among all samples from each corresponding study as target species** | **Maximum proportion classified among all samples from each corresponding study as target genus** |
| --- | --- | --- | --- | --- | --- | --- |
| *A. baumannii* | 99.07% | 99.61% | 97.98% | 99.54% | 97.37% | 99.39% |
| *C. difficile* | 99.40% | 99.40% | 98.95% | 98.95% | 98.37% | 98.37% |
| *E. faecalis* | 99.55% | 99.83% | 99.07% | 99.65% | 98.11% | 98.27% |
| *E. faecium* | 99.30% | 99.95% | 98.74% | 99.79% | 97.7% | 98.97% |
| *K. pneumoniae* | 97.86% | 98.96% | 94.20% | 98% | 94.96% | 97.07% |
| *L. pneumophila* | 99.80% | 100% | 99.61% | 99.98% | 99.80% | 99.84% |
| *L. monocytogenes* | 99.26% | 99.96% | 98.57% | 99.87% | 98.32% | 99.90% |
| *M. tuberculosis*  complex | 99.98% | 100% | 99.98% | 100% | 99.99% | 100% |
| *N. gonorrhoeae* | 99.16% | 100% | 94.96% | 99.99% | 98.72% | 99.98% |
| *P. aeruginosa* | 99.95% | 99.99% | 99.85% | 99.95% | 99.79% | 99.92% |
| *S. enterica* | 99.58% | 99.73% | 98.83% | 99.25% | 98.90% | 99.25% |
| *S. aureus* | 95.01% | 95.39% | 94.57% | 95.35% | 96.10% | 95.15% |
| *T. pallidum* | 93.54% | 100% | 72.74% | 100% | 94.75% | 94.75% |
| *V. cholerae* | 99.59% | 99.83% | 98.90% | 99.74% | 98.70% | 99.82% |

* species in the case of MTB (*Mycobacterium tuberculosis* complex)
