# Supplementary material for: Contaminant DNA in bacterial sequencing experiments is a major source of false genetic variability
Source: BMC Biol. 2020 Mar 2;18:24. doi: 10.1186/s12915-020-0748-z (PMC7053099; doi:10.1186/s12915-020-0748-z)
Supplement: Supplementary file 2 — Additional file 2: Table S2. Evaluation of the performance of Kraken classifying reads at genus and species level for the reference genomes and among all samples of the studies analyzed after excluding the reference genomes from the Kraken database. [file 12915_2020_748_MOESM2_ESM.docx]

**Table S2.** Evaluation of the performance of Kraken classifying reads at genus and species level for the reference genomes after excluding the reference genomes from the Kraken database.

| **Organism** | **Reads classified as target species (Illumina MiSeq; 250bp)** | **Reads classified as target genus (Illumina MiSeq; 250bp)** | **Reads classified as target species (Illumina HiSeq; 100bp)** | **Reads classified as target genus (Illumina HiSeq; 100bp)** |
| --- | --- | --- | --- | --- |
| *A. baumannii* | 99.07% | 99.61% | 97.98% | 99.07% |
| *C. difficile* | 99.39% | 99.39% | 98.95% | 98.95% |
| *E. faecalis* | 90.59% | 92.01% | 89.72% | 91.17% |
| *E. faecium* | 98.27% | 98.92% | 97.53% | 98.62% |
| *K. pneumoniae* | 97.86% | 98.96% | 94.20% | 98.00% |
| *L. pneumophila* | 99.80% | 100% | 99.61% | 99.98% |
| *L. monocytogenes* | 98.49% | 99.21% | 97.76% | 99.10% |
| *N. gonorrhoeae* | 99.16% | 100% | 94.96% | 99.99% |
| *P. aeruginosa* | 99.95% | 99.99% | 99.85% | 99.95% |
| *S. enterica* | 99.58% | 99.73% | 98.83% | 99.25% |
| *S. aureus* | 94.98% | 95.37% | 94.56% | 95.34% |
| *T. pallidum* | 92.96% | 100% | 71.25% | 100% |
| *V. cholerae* | 99.59% | 99.83% | 98.90% | 99.74% |
