# Supplementary material for: Contaminant DNA in bacterial sequencing experiments is a major source of false genetic variability
Source: BMC Biol. 2020 Mar 2;18:24. doi: 10.1186/s12915-020-0748-z (PMC7053099; doi:10.1186/s12915-020-0748-z)
Supplement: Supplementary file 3 — Additional file 3: Table S3. Bracken estimation of the proportion of misclassified and unclassified reads that actually belong to the target organism. [file 12915_2020_748_MOESM3_ESM.docx]

**Table S3.** Proportion of unclassified reads and reads classified at any taxonomic level other that the target genus that are estimated by Bracken to actually belong to the target genus.

| **Organism** | **Percentage of unclassified reads** | **Percentage of unclassified reads reassigned to target genus** | **Percentage of reads assigned to any taxonomic level other than target genus** | **Percentage of reads assigned to taxonomic levels other than the target genus that are reassigned to the target genus** |
| --- | --- | --- | --- | --- |
| *A. baumannii* | 0.59% | 0.06% | 0.52% | 0.01% |
| *C. difficile* | 2.39% | 0.14% | 15.35% | 1.15% |
| *E. faecalis* | 4.69% | 0.92% | 2.53% | 0.44% |
| *E. faecium* | 3.90% | 0.84% | 2.85% | 0.53% |
| *K. pneumoniae* | 2.81% | 0.91% | 7.36% | 2.45% |
| *L. pneumophila* | 0.50% | 0.04% | 0.06% | 0.006% |
| *L. monocytogenes* | 0.12% | 0.05% | 0.67% | 0.28% |
| *M. tuberculosis*  complex | 0.07% | 0.0002% | 0.2% | 0.0006% |
| *N. gonorrhoeae* | 0.03% | 0.007% | 0.06% | 0.01% |
| *P. aeruginosa* | 0.92% | 0.15% | 0.75% | 0.22% |
| *S. enterica* | 0.22% | 0.15% | 3.4% | 2.1% |
| *S. aureus* | 1.33% | 0.6% | 4.05% | 2.04% |
| *T. pallidum* | 0.31% | 0% | 67.32% | 0% |
| *V. cholerae* | 4.37% | 0.23% | 0.97% | 0.07% |
