# Supplementary material for: Contaminant DNA in bacterial sequencing experiments is a major source of false genetic variability
Source: BMC Biol. 2020 Mar 2;18:24. doi: 10.1186/s12915-020-0748-z (PMC7053099; doi:10.1186/s12915-020-0748-z)
Supplement: Supplementary file 4 — Additional file 4: Table S4. Samples of the bacterial dataset with less than 50% of target organism. [file 12915_2020_748_MOESM4_ESM.docx]

**Table S4.** Samples of the bacterial dataset with less than 50% of target organism.

| **Study** | **Sample Accession** | **Percentage of target organism** | **Majoritary classification** |
| --- | --- | --- | --- |
| *Staphylococcus* | ERR033693 | 34.17% | Unclassified (41%) |
| *Staphylococcus* | ERR038670 | 44.74% | *Staphylococcus aureus* (43%) |
| *Staphylococcus* | ERR038673 | 32.07% | *Staphylococcus aureus* (31%) |
| *Staphylococcus* | ERR038675 | 49.30% | *Staphylococcus aureus* (48%) |
| *Staphylococcus* | ERR038682 | 25.64% | Unclassified (44%) |
| *Staphylococcus* | ERR038688 | 36.52% | *Staphylococcus aureus* (35%) |
| *Clostridioides* | SRR3115465 | 49.44% | *Clostridioides difficile* (49%) |
| *Clostridioides* | SRR3115477 | 35.90% | *Clostridioides difficile* (36%) |
| *Clostridioides* | SRR3115503 | 38.51% | *Clostridioides difficile* (38%) |
| *Klebsiella* | ERR025467 | 1.13% | *Enterobacter hormaechei* (10%) |
| *Klebsiella* | ERR025493 | 0.27% | *Escherichia coli* (73%) |
| *Klebsiella* | ERR025508 | 0.33% | *Escherichia coli* (33%) |
| *Klebsiella* | ERR025530 | 0.11% | *Ochrobactrum anthropi* (26%) |
| *Klebsiella* | ERR025533 | 0.77% | *Escherichia coli* (33%) |
| *Klebsiella* | ERR025580 | 0.05% | *Streptococcus agalactiae* (97%) |
| *Klebsiella* | ERR025650 | 0.88% | *Enterobacter xiangfangensis* (14%) |
