# Supplementary material for: Contaminant DNA in bacterial sequencing experiments is a major source of false genetic variability
Source: BMC Biol. 2020 Mar 2;18:24. doi: 10.1186/s12915-020-0748-z (PMC7053099; doi:10.1186/s12915-020-0748-z)
Supplement: Supplementary file 5 — Additional file 5: Table S5. Difference in the number of variant positions within a dataset between the basic and the taxonomic-filtered pipeline. [file 12915_2020_748_MOESM5_ESM.docx]

**Table S5. Difference in the number of non-redundant variant positions within a dataset between the basic and the taxonomic-filtered pipeline.** **E. faecalis* and *E. faecium* are analyzed by separate but belong to the same study. **In the Mozambique study one sample contaminated with 20% of *Mycobacterium sinense* contributed with 72,354 variant positions. The difference observed for the Mozambique study when disregarding this sample was of 927 vSNP positions (20.1%).

| **Study** | **Dataset** | **Difference in fSNP positions** | **Difference in vSNP positions** |
| --- | --- | --- | --- |
| *A. baumannii* | *Bacterial dataset* | 600 (0.15%) | 129 (1.52%) |
| *C. difficile* | *Bacterial dataset* | 3,624 (5.84%) | 1,437 (10.53%) |
| *E. faecalis** | *Bacterial dataset* | 3,676 (1.94%) | 635 (5.61%) |
| *E. faecium** | *Bacterial dataset* | 492 (0.25%) | 195 (0.89%) |
| *K. pneumoniae* | *Bacterial dataset* | 6,828 (1.17%) | 68,246 (23.93%) |
| *L. pneumophila* | *Bacterial dataset* | 222 (0.12%) | 157 (1.37%) |
| *L. monocytogenes* | *Bacterial dataset* | 68 (0.03%) | 53 (0.71%) |
| *N. gonorrhoeae* | *Bacterial dataset* | 0 (0%) | 2 (0.03%) |
| *P. aeruginosa* | *Bacterial dataset* | 299 (0.09%) | 276 (2.38%) |
| *S. enterica* | *Bacterial dataset* | 813 (2.92%) | 645 (19.11%) |
| *S. aureus* | *Bacterial dataset* | 3,652 (2.14%) | 2,082 (3.57%) |
| *T. pallidum* | *Bacterial dataset* | 26 (4.47%) | 252 (41.17%) |
| *V. cholerae* | *Bacterial dataset* | 2,190 (0.55%) | 220 (1.84%) |
| Kwazulu-Natal | *MTB dataset* | 1 (0%) | 240 (9.22%) |
| Nigeria | *MTB dataset* | 3 (0.32%) | 170 (37.11%) |
| Belarus | *MTB dataset* | 0 (0%) | 71 (3.42%) |
| Mozambique | *MTB dataset* | 39 (0.32%) | 71,309 (92.65%) ** |
| High-depth sequencing | *MTB dataset* | 0 (0%) | 161 (3.40%) |
| Sputum capture-sequencing | *MTB dataset* | 124 (2.19%) | 28,452 (95.11%) |
| Sputum direct-sequencing | *MTB dataset* | 24 (0.49%) | 3,686 (83.79%) |
| MGIT sequencing | *MTB dataset* | 19 (0.11%) | 517 (18%) |
