# Supplementary material for: Contaminant DNA in bacterial sequencing experiments is a major source of false genetic variability
Source: BMC Biol. 2020 Mar 2;18:24. doi: 10.1186/s12915-020-0748-z (PMC7053099; doi:10.1186/s12915-020-0748-z)
Supplement: Supplementary file 6 — Additional file 6: Table S6. Proportion of fSNPs removed per sample in the bacterial dataset. (DOCX 6 kb) [file 12915_2020_748_MOESM6_ESM.docx]

**Table S6 -** Proportion of fSNPs removed per sample in the *bacterial dataset.*

| **Study** | **Proportion of fSNPs removed by the taxonomic filter (median)** |
| --- | --- |
| *A. baumannii* | 0.22% |
| *C. difficile* | 0.72% |
| *E. faecalis* | 0.52% |
| *E. faecium* | 0.16% |
| *K. pneumoniae* | 0.81% |
| *L. pneumophila* | 0.00% |
| *L. monocytogenes* | 0.03% |
| *N. gonorrhoeae* | 0.00% |
| *P. aeruginosa* | 0.00% |
| *S. enterica* | 5.58% |
| *S. aureus* | 1.89% |
| *T. pallidum* | 0.00% |
| *V. cholerae* | 0.32% |
