# Supplementary material for: Contaminant DNA in bacterial sequencing experiments is a major source of false genetic variability
Source: BMC Biol. 2020 Mar 2;18:24. doi: 10.1186/s12915-020-0748-z (PMC7053099; doi:10.1186/s12915-020-0748-z)
Supplement: Supplementary file 7 — Additional file 7: Figure S1. Effects of contaminations and taxonomic filtering in variant calling. [file 12915_2020_748_MOESM7_ESM.pdf]

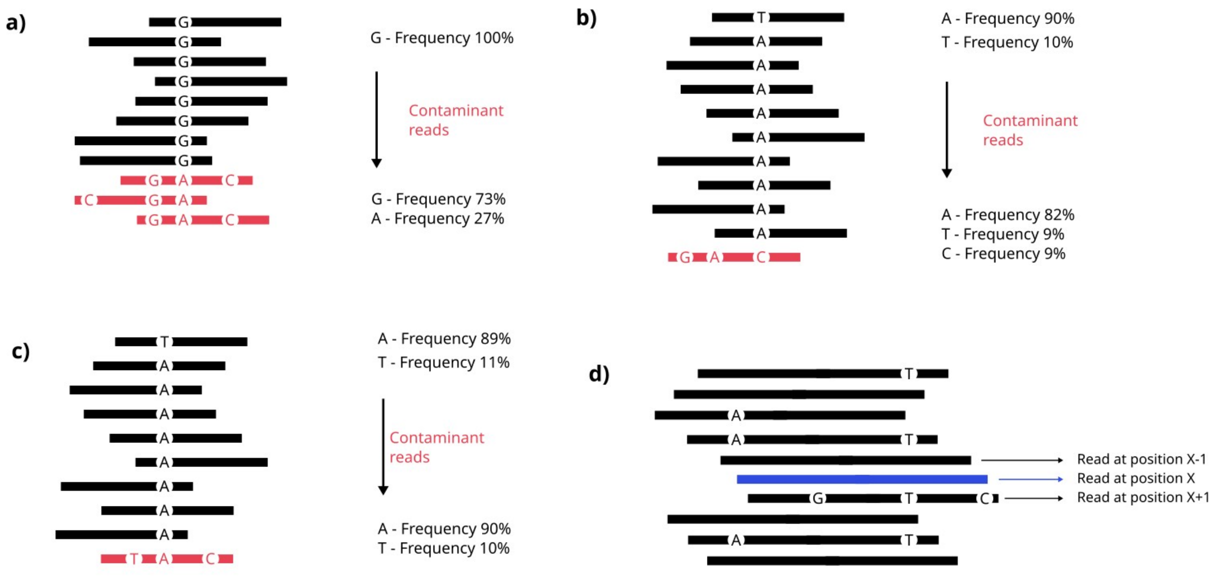

Figure S1. Effects of contaminating reads and taxonomic filtering in variant calling. In this figure we exemplified possible effects of contaminant reads (in red) in variant calling (**a-c**) and the inability of Kraken classifying some reads (**d**). **a)** Contaminant reads make G to be called as vSNP instead of fSNP an introduce a false positive vSNP (A) **b)** Contaminant reads make A to be called as vSNP instead of a fSNP and make one vSNP (T) to be lost due to SNP calling cutoffs (10% frequency for vSNPs). **c)** Contaminant reads make A to be called as fSNP instead of a vSNP. **d)** The read coloured in blue cannot be classified by Kraken up to the level of genus. This occurs despite the fact that this read is 100% identical to the reference and that surrounding reads, with several SNPs, can be classified even to the level of species.
