# Supplementary material for: Contaminant DNA in bacterial sequencing experiments is a major source of false genetic variability
Source: BMC Biol. 2020 Mar 2;18:24. doi: 10.1186/s12915-020-0748-z (PMC7053099; doi:10.1186/s12915-020-0748-z)
Supplement: Supplementary file 8 — Additional file 8 :Figure S2. Contaminations can lead to incorrect calls across the M. tuberculosis genome. [file 12915_2020_748_MOESM8_ESM.pdf]

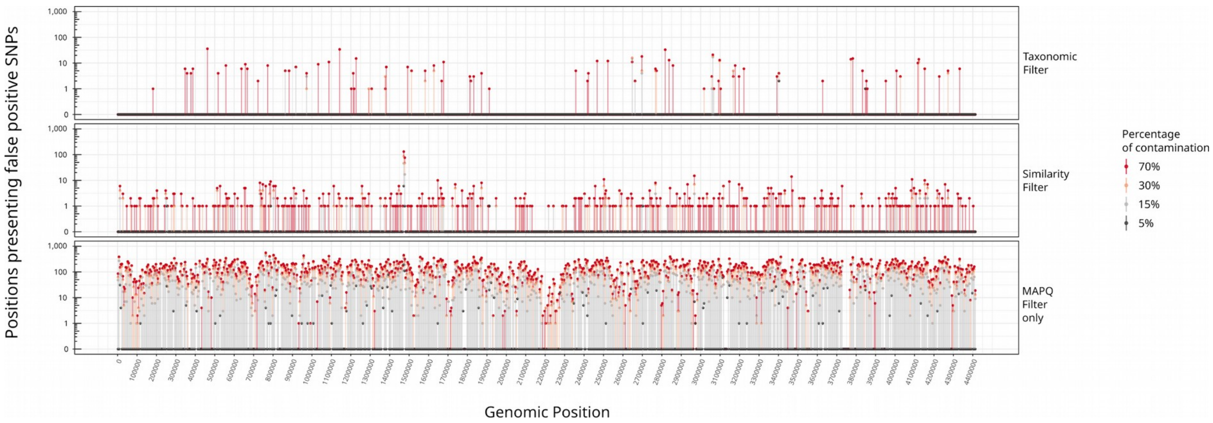

Figure S2. Contaminations can lead to incorrect calls across the *M. tuberculosis* genome. Number of positions (in 1,000 bp windows) with false positive SNPs arising from 5%, 15%, 30% and 70% contaminations with different organisms in mock contaminated MTB WGS samples.
