# Supplementary material for: Contaminant DNA in bacterial sequencing experiments is a major source of false genetic variability
Source: BMC Biol. 2020 Mar 2;18:24. doi: 10.1186/s12915-020-0748-z (PMC7053099; doi:10.1186/s12915-020-0748-z)
Supplement: Supplementary file 9 — Additional file 9: Table S7. Evaluation of false drug resistance predictions in mock contaminated samples. [file 12915_2020_748_MOESM9_ESM.docx]

**Table S7.** Evaluation of false drug resistance predictions in mock contaminated samples.

| **Contaminant** | **Proportion** | **Mutation** | **Gene; Drug** |
| --- | --- | --- | --- |
| *R. mucilaginosa* | Starting at 5% | 1473247 C>A (HC, new) | rrs (16S); AMK/KAN/CPR |
|  |  | 1473329 G>T (HC; described) |  |
| *A. oris* | Starting at 15% | 761154 T>A (LC, new) | rpoB; RMP |
|  |  | 761155 C>G (HC, described) |  |
| *M. abscessus* | Starting at 15% | 761098 G>C (LC, described) | rpoB; RMP |
| *M. avium* | Starting at 15% | 6738 C>A (HC, described) | gyrB; FQ |
|  |  | 6742 A>G (HC, new) |  |
|  | Starting at 70% | 761098 G>C (LC, described) | rpoB; RMP |
| *M. chimaera* | Starting at 15% | 6742 A>G (HC, new) | gyrB; FQ |
|  | Starting at 30% | 1834852 C>G (LC, new) | rpsA; PZA |
|  |  | 4243245 G>C (LC, new) | embA; EMB |
|  |  | 4247729 G>C (HC, new) | embB; EMB |
|  |  | 4247730 G>C (HC, described) |  |
|  | Starting at 70% | 2289100 T>G (HC, new) | pncA; PZA |
| *M. fortuitum* | Starting at 15% | 761111 C>T (LC, new) | rpoB; RMP |
|  | Starting at 30% | 1834852 C>G (LC, new) | rpsA; PZA |
|  | Starting at 70% | 761098 G>C (LC, described) | rpoB; RMP |
|  |  | 1918494 T>C (LC, new) | tlyA; CPR |
| *M. kansasii* | Starting at 15% | 761098 G>C (LC, described) | rpoB; RMP |
|  |  | 1834852 C>G (LC, new) | rpsA; PZA |
|  | Starting at 30% | 781687 | rpsL; SM |
|  | Starting at 70% | 1918494 T>C (LC, new) | tlyA; CPR |

False drug-resistance predictions according to high confidence (HC) and low confidence (LC) known mutations in the PhyResSE catalog. SNPs tagged as *new* correspond to undescribed variants produced in positions known to harbour drug-resistance conferring mutations.AMK (Amikacin); CPR (Capreomycin); EMB (Ethambutol); ETH (Ethionamide); FQ (Fluoroquinolones); INH (Isoniazid); KAN (Kanamycin); LZD (Linezolid); PAS (Para-aminosalicylic acid); PZA (Pyrazinamide); RMP (Rifampicin); SM (Streptomycin).
