# Supplementary material for: Contaminant DNA in bacterial sequencing experiments is a major source of false genetic variability
Source: BMC Biol. 2020 Mar 2;18:24. doi: 10.1186/s12915-020-0748-z (PMC7053099; doi:10.1186/s12915-020-0748-z)
Supplement: Supplementary file 10 — Additional file 10: Table S8. Genomic regions (1000 bp windows) with a coverage decrease greater than 1X after taxonomic filtering in 984 samples of the MTB dataset with more than 99% of reads classified as MTB. [file 12915_2020_748_MOESM10_ESM.docx]

**Table S8** - Genomic regions (1,000 bp windows) with a coverage decrease greater than 1X after taxonomic filtering for 984 samples of the *MTB dataset* with more than 99% of reads classified as MTB.

| **Region** | **Sequencing depth difference (mean)** | **Annotation** |
| --- | --- | --- |
| 1472000:1472999 | 113.82 | *rrs* |
| 1475000:1475999 | 20.52 | *rrl* |
| 1473000:1473999 | 12.56 | *rrs* |
| 1476000:1476999 | 8.53 | *rrl* |
| 1474000:1474999 | 3.47 | *rrl* |
| 1471000:1471999 | 2 | *murA* |
| 3705000:3705999 | 1.66 | *sdhA* |
| 1649000:1649999 | 1.28 | Rv1461 |
| 932000:932999 | 1.13 | Intergenic |
