# Supplementary material for: Contaminant DNA in bacterial sequencing experiments is a major source of false genetic variability
Source: BMC Biol. 2020 Mar 2;18:24. doi: 10.1186/s12915-020-0748-z (PMC7053099; doi:10.1186/s12915-020-0748-z)
Supplement: Supplementary file 11 — Additional file 11: Table S9. Top ten genomic regions (1000 bp windows) with greater coverage decrease after applying the similarity filter in 984 samples of the MTB dataset with more than 99% of reads classified as MTB. [file 12915_2020_748_MOESM11_ESM.docx]

**Table S9** - Top ten genomic regions (1,000 bp windows) with greater coverage decrease after applying the similarity filter for 984 samples of the *MTB dataset* with more than 99% of reads classified as MTB.

| **Region** | **Sequencing depth difference (mean)** | **Annotation** |
| --- | --- | --- |
| 2266000:2266999 | 49.45 | Rv2019,Rv2020c,Rv2021c |
| 336000:336999 | 26.73 | *PE-PGRS3,PE-PGRS4* |
| 4383000:4383999 | 23.86 | *Rv3897c,Rv3898c* |
| 2137000:2137999 | 19.74 | *Rv1887,Rv1888c* |
| 3119000:3119999 | 19.27 | *Rv2813. Intergenic region(Rv2813-Rv2814c)* |
| 3750000:3750999 | 18.89 | *Rv3347c (PPE Family protein)* |
| 467000:467999 | 18.35 | *Rv0387c, Rv0388c (PPE Family protein)* |
| 2421000:2421999 | 17.46 | Rv2159c, Rv2160c |
| 3843000:3843999 | 17.16 | Rv3426 (PPe Family protein), Rv3427c |
| 3296000:3296999 | 17.13 | Rv2946c, Rv2947c (pks15) |
