# Supplementary material for: Contaminant DNA in bacterial sequencing experiments is a major source of false genetic variability
Source: BMC Biol. 2020 Mar 2;18:24. doi: 10.1186/s12915-020-0748-z (PMC7053099; doi:10.1186/s12915-020-0748-z)
Supplement: Supplementary file 12 — Additional file 12: Table S10. Reference genomes of the bacterial dataset. [file 12915_2020_748_MOESM12_ESM.docx]

**Table S10.** Reference genomes of the *bacterial dataset*.

| **Dataset** | **Organism** | **Reference** |
| --- | --- | --- |
| *Acinetobacter* | *A. baumannii* | CP_000521.1 |
| *Clostridium* | *C. difficile* | NC_009089.1 |
| *Enterococcus* | *E. faecalis* | NC_004668.1 |
| *Enterococcus* | *E. faecium* | NC_017960.1 |
| *Klebsiella* | *K. pneumoniae* | AP006725.1 |
| *Listeria* | *L. monocytogenes* | NC_003210.1 |
| *Legionella* | *L. pneumophila* | NC_002942.5 |
| *Neisseria* | *N. gonorrhoeae* | GCF_900087815.1 |
| *Pseudomonas* | *P. aeruginosa* | NC_002516.2 |
| *Staphylococcus* | *S. aureus* | NC_007795.1 |
| *Salmonella* | *S. enterica* | NC_003197.2 |
| *Treponema* | *T. pallidum* | NZ_CP003679.1 |
| *Vibrio* | *V. cholerae* | NC_002505.1 & NC_002506.1 |
