# Supplementary material for: Contaminant DNA in bacterial sequencing experiments is a major source of false genetic variability
Source: BMC Biol. 2020 Mar 2;18:24. doi: 10.1186/s12915-020-0748-z (PMC7053099; doi:10.1186/s12915-020-0748-z)
Supplement: Supplementary file 13 — Additional file 13: Figure S3. Implementation of the similarity mapping filter. [file 12915_2020_748_MOESM13_ESM.pdf]

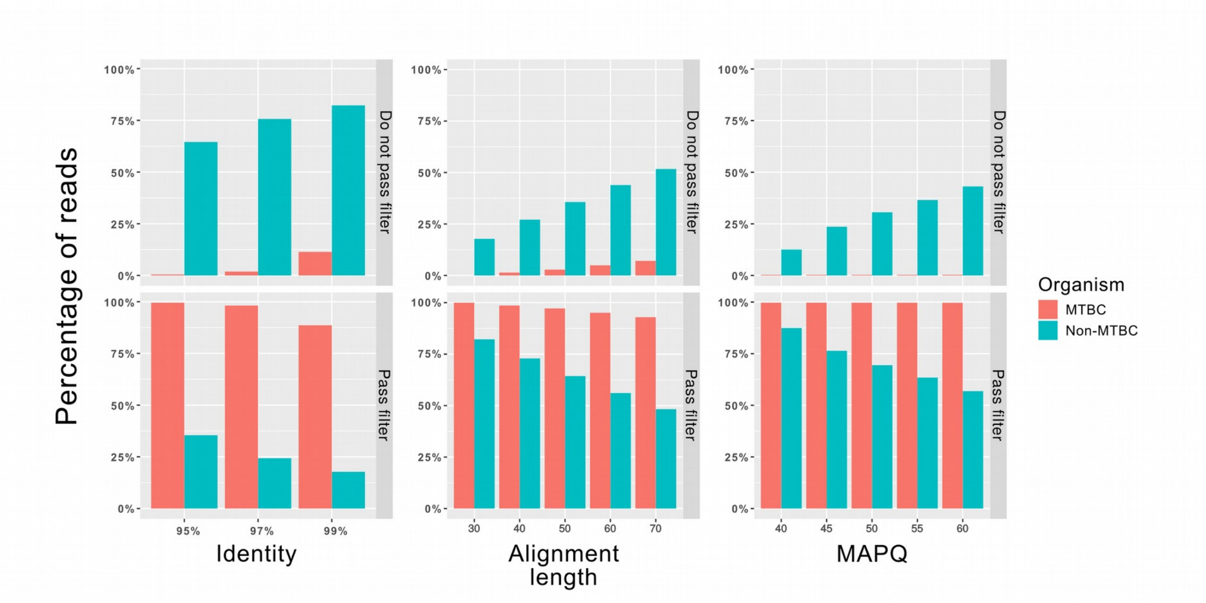

Figure S3. Implementation of the similarity mapping filter. Simulated reads from organisms of the *Mycobacterium tuberculosis* complex (MTBC) and non-MTBC organisms were mapped to the MTB reference genome and alignments were then filtered using different parameters. Looking for the parameters that maximized the removal of non-MTBC mappings without filtering true MTBC alignments, we implemented a similarity mapping filter that removes alignments with identities, lengths and mapping qualities below 97%, 40 bp and 60 respectively.
