# Supplementary material for: Contaminant DNA in bacterial sequencing experiments is a major source of false genetic variability
Source: BMC Biol. 2020 Mar 2;18:24. doi: 10.1186/s12915-020-0748-z (PMC7053099; doi:10.1186/s12915-020-0748-z)
Supplement: Supplementary file 14 — Additional file 14: Table S11. Non-MTB species included in the simulated sequencings to evaluate the impact of contaminations in MTB WGS samples. [file 12915_2020_748_MOESM14_ESM.docx]

**Table S11.** Non-MTB species included in the simulated sequencing experiments to evaluate the impact of contaminations in MTB WGS samples

| **Species** | **RefSeq Assembly Accession_Version** |
| --- | --- |
| *Homo sapiens* | GRCh38, Ensembl release 81 |
| *Acinetobacter baumannii* | GCF_000746645.1_ASM74664v1 |
| *Actinomyces oris* | GCF_001553935.1_ASM155393v1 |
| *Atopobium parvulum* | GCF_000024225.1_ASM2422v1_V2 |
| *Bacillus cereus* | GCF_000007825.1_ASM782v1 |
| *Bacillus thuringiensis* | GCF_000497525.1_ASM49752v2 |
| *Bifidobacterium dentium* | GCF_001042595.1_ASM104259v1 |
| *Campylobacter concisus* | GCF_003049735.1_ASM304973v1 |
| *Corynebacterium pseudodiphtheriticum* | GCF_000688415.1_ASM68841v1 |
| *Enterobacter cloacae* | GCF_000025565.1_ASM2556v1 |
| *Enterococcus faecalis* | GCF_000007785.1_ASM778v1 |
| *Eubacterium aggregans* | GCF_900107815.1_IMG-taxon_2642422588 |
| *Fusobacterium sp.* | GCF_900015295.1_clos_1_1 |
| *Gemella sp. (oral taxon)* | GCF_001553915.1_ASM155391v1 |
| *Granulicatella elegans* | GCF_000162475.2_Gran_ele_ATCC_700633_V2 |
| *Klebsiella pneumoniae* | GCF_000240185.1_ASM24018v2 |
| *Micrococcus luteus* | GCF_000023205.1_ASM2320v1 |
| *Mycobacterium abscessus* | GCF_000069185.1_ASM6918v1 |
| *Mycobacterium avium* | GCF_000240505.1_ASM24050v2 |
| *Mycobacterium chimaera* | GCF_900116695.1_ASM90011669v1 |
| *Mycobacterium fortuitum* | GCF_001307545.1_ASM130754v1 |
| *Mycobacterium haemophilum* | GCF_000340435.2_ASM34043v3 |
| *Mycobacterium kansasii* | GCF_000157895.3_ASM15789v2 |
| *Mycobacterium phlei* | GCF_001583415.1_ASM158341v1 |
| *Mycobacterium sinense* | GCF_001667945.1_ASM166794v1 |
| *Mycobacterium smegmatis* | GCF_000015005.1_ASM1500v1 |
| *Negativicoccus massiliensis* | GCF_900155405.1_PRJEB18760 |
| *Neisseria perflava* | GCF_002863305.1_ASM286330v1 |
| *Nocardia brasiliensis* | GCF_000250675.2_ASM25067v3 |
| *Nocardia farcinica* | GCF_001182745.1_NCTC11134 |
| *Nocardia nova* | GCF_000523235.1_ASM52323v1 |
| *Porphyromonas sp. (oral taxon)* | GCF_000292995.1_Psp279F0450v1.0 |
| *Prevotella sp. (oral taxon)* | GCF_000163055.2_ASM16305v2 |
| *Propionibacterium sp. (oral taxon)* | GCF_001717565.1_ASM171756v1 |
| *Pseudomonas aeruginosa* | GCF_000006765.1_ASM676v1 |
| *Pseudomonas fluorescens* | GCF_000237065.1_ASM23706v1 |
| *Rothia mucilaginosa* | GCF_000011025.1_ASM1102v1 |
| *Staphylococcus aureus* | GCF_000013425.1_ASM1342v1 |
| *Streptococcus mitis* | GCF_000027165.1_ASM2716v1 |
| *Streptococcus oralis* | GCF_002871315.1_ASM287131v1 |
| *Streptococcus parasanguinis* | GCF_000164675.2_ASM16467v2 |
| *Streptococcus pneumoniae* | GCF_000007045.1_ASM704v1 |
| *Streptococcus salivarius* | GCF_000785515.1_ASM78551v1 |
| *Veillonella sp. (oral taxon)* | GCF_000221605.1_ASM22160v1 |
| *Yersinia enterocolitica* | GCF_000009345.1_ASM934v1 |
